# The mediating role of physical self-perception and the moderating effect of gender in the association between physical activity and psychological well-being among young recreational participants

**DOI:** 10.3389/fpsyg.2026.1886525

**Published:** 2026-08-19

**Authors:** Ozlem Ekizoglu, Sibel Tetik, Omer Zambak, Canan Sayin Temur, Yusuf Can, Utku Isik, Mustafa Can Koc

**Affiliations:** 1 Faculty of Sports Sciences, Erzincan Binali Yildirim University, Erzincan, Türkiye; 2 Faculty of Sports Sciences, Gumushane University, Gumushane, Türkiye; 3 Faculty of Sports Sciences, Ankara Yildirim Beyazit University, Ankara, Türkiye; 4 Faculty of Sports Sciences, Istanbul Gelisim University, Istanbul, Türkiye; 5 Faculty of Sports Sciences, Ege University, Izmir, Türkiye; 6 Faculty of Sports Sciences, Mersin University, Mersin, Türkiye

**Keywords:** gender moderation, physical activity, physical self-perception, psychological well-being, recreational sports

## Abstract

**Background/objective:**

Psychological well-being disorders are increasingly prevalent among young people. While physical activity is known to benefit psychological well-being, the mechanisms—particularly the role of physical self-perception and gender in recreational context remain underexplored. Grounded in the Conservation of Resources (COR) Theory, this study investigates the mediating role of physical self-perception in the relationship between recreational physical activity and psychological well-being and examines whether gender moderates this mediation among youth aged 15–24.

**Methods:**

A descriptive-relational survey design was employed. The sample comprised 468 young adults (58.5% male, 41.5% female; M_age = 20.08) who regularly participated in recreational physical activities. Data were collected using the International Physical Activity Questionnaire-Short Form (IPAQ-SF), the Physical Self-Perception Profile (PSPP), and the Warwick-Edinburgh Mental Well-being Scale (WEMWBS). Mediation and moderation analyses were conducted using PROCESS macro-Models 4 and 7.

**Results:**

The direct effect of physical activity on psychological well-being was not significant. However, physical self-perception fully mediated the relationship between physical activity and psychological well-being (indirect effect: *β* = 0.219, 95% CI [0.151, 0.290]). Among the sub-dimensions of physical self-perception, body attractiveness had the strongest mediating effect. Moderation analysis revealed that gender did not significantly moderate the mediation pathways (a path: *p* = 0.156; b path: *p* = 0.954). The mediation mechanism operated with similar strength in both males and females.

**Conclusion:**

Recreational physical activity improves psychological well-being entirely through the enhancement of physical self-perception, supporting the COR theory that physical activity acts as a resource investment. This mechanism is universal across genders in recreational settings. Interventions aiming to improve youth psychological well-being should not only promote physical activity but specifically focus on fostering positive physical self-perception and body image in inclusive, non-judgmental environments.

## 1 Introduction

Mental health disorders have become a global health issue today. Depression, anxiety, and other mental health issues are becoming more common, especially among young people (Fu et al., 2025). According to the latest reports from the World Health Organization, mental health issues are a major cause of death and illness among young people aged 15 to 24 (United Nations, 2015). This period is a time when important developmental tasks such as identity formation, academic pressure, social adjustment, and future planning are fulfilled, and these factors can make young people particularly vulnerable in terms of mental health.

Participation in physical activity has emerged as a promising intervention for the prevention and treatment of mental health issues. Numerous studies have demonstrated that regular physical activity reduces the symptoms of depression, anxiety, stress, and other mental health issues (Laurier et al., 2024; Chiang et al., 2024). However, the mechanisms by which physical activity affects mental health are not yet fully understood. There is limited information regarding the psychological and physiological pathways of this relationship, the factors that regulate it, and the functioning of these mechanisms in the context of recreational activity participation.

The theoretical foundation of this research is the Conservation of Resources (COR) Theory. The COR theory is a stress and coping theory developed by Hobfoll (1989) and later expanded by Hobfoll (2001). According to the theory, individuals acquire, preserve, and expand emotional, cognitive, and social resources to cope with life stress. The preservation and renewal of these resources enhance psychological resilience and adaptability, thereby safeguarding individuals against adverse emotional outcomes (Hobfoll, 2011). In the context of COR theory, physical activity can be conceptualized as a behavioral resource investment. Wang et al. (2025) conducted a study illustrating the chain of physical activity → self-efficacy (cognitive resource) → emotion regulation (emotional resource) → mental health. This model illustrates that physical activity initiates a spiral of resource accumulation, which in turn supports psychological well-being. From the COR perspective, physical self-perception can be an important personal resource that can be developed and maintained through physical activity, and that can help individuals gain and protect resources (Howard, 2019). When young people participate in fun physical activities, they have positive experiences regarding physical fitness, strength, body appearance, and overall physical condition. These experiences likely enhance physical self-perception and serve as a resource that improves mental health.

Physical self-perception and physical self-esteem, despite representing different dimensions of self-esteem, are often used interchangeably in the literature. Physical self-perception is conceptualized as a broad and multidimensional construct encompassing individuals’ perceptions, feelings, and evaluative judgments about their physical selves, including dimensions such as sport competence, physical condition, body attractiveness, and physical strength (Fox and Corbin, 1989; Marsh, 1990). In contrast, physical self-esteem is a more limited construct that encompasses self-evaluations related to physical activity, sports competence, appearance, and weight (Fernández-Bustos et al., 2019). In other words, physical self-perception refers to how the body is perceived, while physical self-esteem refers to how these perceptions reflect the individual’s self-worth. This conceptual distinction is important for understanding the relationship between physical activity and psychological well-being.

A review of the current literature reveals that the mediating role of physical self-perception in the relationship between physical activity and psychological well-being has been inadequately explored, particularly among adolescents and in the context of recreational activities. The systematic review and meta-analysis conducted by Fu et al. (2025) emphasized that the mediation mechanisms in this relationship have not yet been fully elucidated. While the mediating function of physical self-esteem is substantiated within the Exercise and Self-Esteem Model (Sonstroem and Morgan, 1989), subsequent empirical investigations have revealed indirect pathways from physical activity to psychological well-being via physical self-perceptions (e.g., McAuley et al., 2005; Elavsky et al., 2005). However, these studies predominantly concentrate on university students or adult cohorts, insufficiently exploring the context of recreational physical activity. Consequently, the manner in which physical activity alters individuals’ perceptions of their bodies and the subsequent impact of these perceptual changes on mental health remains insufficiently elucidated. The second significant gap pertains to the regulatory role of gender. Luo et al. (2025) and Tian et al. (2022) have demonstrated that gender serves as a moderating factor in the relationship between physical activity and mental health. However, these studies have predominantly concentrated on the direct relationship between physical activity and mental health, neglecting intermediary mechanisms such as physical self-perception in their models. Consequently, there is no empirical evidence regarding how gender influences indirect pathways through physical self-perception. However, women and men may attribute varying degrees of importance to different aspects of physical self-perception (e.g., appearance, muscle strength, endurance); this may result in gender-specific differences in the impact of physical activity on mental health. Thirdly, these mechanisms have not yet been sufficiently tested in the context of recreational physical activity. Murphy et al. (2020) demonstrated a positive correlation between recreational sports participation and mental health; however, they did not investigate the underlying psychological mechanisms of this relationship. Unlike organized sports or structured exercise programs, the social, motivational, and psychological characteristics of recreational activities can shape these relationships in distinct ways (Tsai et al., 2015).

Therefore, considering the unique structure of the recreational context, it is necessary to address both mediating and regulatory mechanisms together. The current study significantly contributes to addressing the identified gaps in the literature by providing practical insights into optimizing enjoyable physical activities as a vehicle for youth mental health support. The findings offer a strategic framework for universities, athletic centers, and healthcare providers to develop precision-based interventions aimed at fostering psychological resilience and improving psychological well-being outcomes among young adults.

In conclusion, although the current literature has significantly elucidated the relationship between physical activity and psychological well-being, there are significant gaps regarding (i) the mediating role of physical self-perception, (ii) the regulatory effect of gender on this mediating process, and (iii) how these mechanisms operate within the context of recreational physical activity. Consequently, to comprehensively comprehend the impact of physical activity on psychological well-being, it is essential to examine the mediating role of physical self-perception and the regulatory influence of gender within this mediation process. Based on the findings of previous studies, the first hypothesis of this study was formulated as follows:

*H1*:Physical activity has a direct positive effect on psychological well-being.

In the literature, physical activity has generally been regarded as a broad concept, with no distinction made between organized sports, exercise programs, and recreational activities. Recreational physical activities—such as futsal, street basketball, and cycling—have different characteristics from organized sports. Recreational activities are less structured, more social, and guided by the participants’ own motivations. Murphy et al. (2020) conducted a study that identified a positive correlation between recreational sports participation and mental health; however, the underlying mechanisms of this relationship remain insufficiently elucidated.

In recent years, the participation rates of young people in recreational physical activities have been increasing. There is limited research on how participation in these activities affects the mental health of young people, how long these effects last, and what factors strengthen or weaken these effects (Zulyniak et al., 2020; Ma et al., 2020). Literature still has gaps when it comes to how recreational activities affect psychological structures like physical self-esteem and how this affects mental health.

*H2*: Physical self-perception mediates the relationship between physical activity and psychological well-being.

Physical self-perception is how individuals think and evaluate their physical abilities, appearance, and competence (Fox and Corbin, 1989). This concept, unlike general self-esteem, is a domain-specific self-evaluation in the physical realm and is multidimensional. Chen et al. (2025) conducted a study demonstrating that physical self-perception partially mediates the relationship between physical activity and quality of life. Zhu et al. (2025) similarly discovered that physical self-esteem serves as a mediating factor between physical activity and prosocial behavior.

There is evidence that self-esteem has direct effects on psychological well-being. Individuals with high self-esteem report better psychological well-being outcomes, lower levels of depression and anxiety, and greater overall life satisfaction (Pan et al., 2025). However, the model of physical activity → physical self-perception → psychological well-being has not been fully tested among adolescents, particularly in the context of recreational activities.

*H3*: Gender moderates the mediating relationship between physical activity, physical self-perception, and psychological well-being.

Gender emerges as a significant variable in the relationship between physical activity and psychological well-being. Luo et al. (2025) conducted a meta-analysis that demonstrated the regulation of the relationship between gender, physical activity, and psychological well-being. Likewise, Tian et al. (2022) indicated that gender serves as a moderator in the relationship between physical activity and anxiety, while Fuentealba-Urra et al. (2021) demonstrated that gender exerts a regulatory influence on the relationship between physical activity and subjective well-being. These findings indicate that women may be more sensitive to the effects of physical activity on psychological well-being compared to men.

Recent studies have sought to examine the mechanisms underlying this moderating effect in greater detail. For instance, Zhao et al. (2025) conducted a study involving 4,763 students, revealing that gender significantly influenced the relationship between physical activity and emotional regulation strategies. Likewise, Liu and Liu (2025) reported that gender played a significant moderating role in the relationship between physical activity and self-esteem among a sample of 712 university students, with a stronger effect observed in females. Jankauskiene and Baceviciene (2022) demonstrated that subjective well-being mediates the relationship between physical activity and anxiety, while gender moderates this mediating process. In an earlier study, Haugen et al. (2011) stated that higher levels of physical activity strengthen the physical self-esteem in both male and female adolescents, resulting in positive effects on overall self-worth.

When these studies are evaluated together, it becomes evident that gender is not merely a demographic variable but a significant regulatory factor influencing the impact of physical activity on psychological well-being. However, the literature does not yet sufficiently clarify how the various dimensions of physical self-perception differ by gender and how this differentiation structures the relationship between physical activity and psychological well-being. The current findings suggest that physical self-perception may exert a more significant influence on psychological well-being in women, whereas in men, the impact of physical activity on psychological well-being may be more direct.

In this context, examining whether gender plays a regulatory role in the relationship between physical activity, body image, and psychological well-being can elucidate how the dimensions of body image differ by gender, thereby contributing theoretically and empirically to literature.

This study looks at the links between recreational physical activity, self-perception of physical health, and psychological well-being among young people aged 15 to 24. The primary objective of the research is to examine the mediating role of physical self-perception in the impact of physical activity on psychological well-being, as well as the moderating role of gender in this mediating relationship. First, it will explain the mechanisms behind this relationship by testing the hypothesis that physical self-perception plays a full mediating role between physical activity and psychological well-being. Second, it will help develop gender-specific intervention strategies by examining how gender organizes this mediating relationship. Thirdly, by examining these mechanisms, it will help to understand the impact of more accessible and sustainable types of physical activity on the psychological well-being of young people in their daily lives.

## 2 Materials and methods

### 2.1 Research model

**Figure 1:**
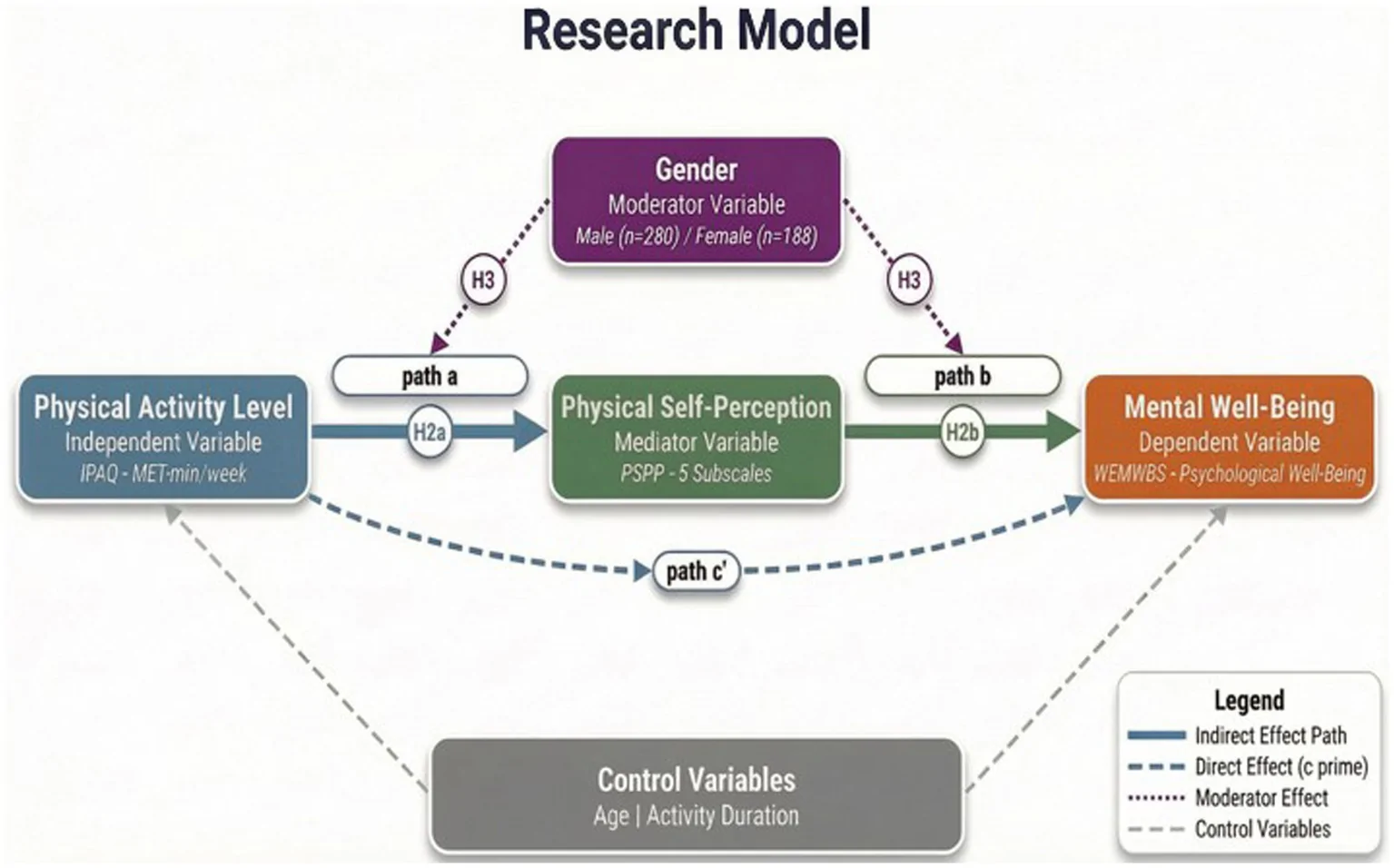
Research model.

The current study was created using a quantitative research framework with a descriptive-relational survey methodology. The conceptual model, developed to determine the role of physical activity on mental well-being, is shown in Figure 1. In this model, the dependent variable is Mental Well-Being, as measured by the Warwick-Edinburgh Mental Well-being Scale (WEMWBS). The independent variable is Physical Activity Level, measured in MET-min/week using the International Physical Activity Questionnaire (IPAQ). The model posits that Physical Self-Perception, measured by the Physical Self-Perception Profile (PSPP) with its 5 subscales, acts as a mediator in this relationship. Furthermore, Gender is examined as a potential moderator of the mediation pathways, while Age and Activity Duration are included as control variables.

### 2.2 Population and sample

Young people aged 15–24 participated in the study. The participants fall within the “youth period” defined by the United Nations for statistical purposes (United Nations, 2015). The sample of the study consists of individuals who regularly participate in recreational physical activities (such as futsal, street basketball, cycling, etc.). Recreational activities, unlike organized sports, are less structured, driven by social motivations, and organized according to the participants’ own preferences. Using a convenience sampling strategy, participants were recruited on-site at various recreational sports venues including university sports centers, public sports facilities, and community recreation centers. Recruitment occurred during peak recreational sports hours to maximize participant availability. Trained research assistants approached individuals at these venues, explained the study, and invited eligible individuals to participate.

The sample size of the study was determined using the G*Power 3.1 software (Faul et al., 2007). For correlation analysis, a small effect size (*ρ* = 0.15), a significance level of *α* = 0.05, and a power level of 80% (1−β) have been targeted. 05, and an 80% power level (1−β) have been targeted. As a result of the calculation with these parameters, the minimum sample size was determined to be 343. In this study, we reached *N* = 468 participants, which is more than sufficient to meet the target. A total of *N* = 468 participants were included in the study. The average age of the participants is 20.08 years (SD = 2.39), and their ages range from 15 to 24 years. The sample consists of 58.5% men (*n* = 274) and 41.5% women (*n* = 194). The average duration of participation in recreational physical activities among the participants is 24.32 months (SD = 10.06), ranging from a minimum of 1 month to a maximum of 59 months.

The criteria for inclusion in the research are as follows: (1) being between 15 and 24 years old; (2) having participated in recreational physical activities at least once a week in the past 6 months; (3) voluntarily agreeing to participate in the research; (4) possessing the cognitive ability to accurately complete the survey forms.

To not participate in the study, the following conditions must be met: (1) being a professional or licensed athlete; (2) having a chronic illness that prevents physical activity; (3) having received a psychiatric diagnosis or being on psychiatric medication within the last 6 months; (4) not having fully completed the survey forms.

### 2.3 Data collection tools

Data were collected using the personal information form prepared by the researchers, the International Physical Activity Questionnaire-Short Form, Physical Self-Perception Profile – (PSPP), and Warwick-Edinburgh Mental Wellbeing Scal- Short Form (WEMWB-SF).

*Data collection process*: The data was collected between October and December 2024. Participants were approached in areas where recreational physical activities are conducted (such as artificial turf complexes, sports parks, bike paths, etc.). The purpose, procedure, and confidentiality guaranties of the research were explained to the participants both in writing and verbally. For adult participants (aged 18 and above), written informed consent was obtained before participation. For participants under the age of 18, written informed consent was obtained from their parents or legal guardians, and the minor participants themselves provided written assent confirming their voluntary agreement to participate. The informed consent and assent forms clearly described the study’s purpose, procedures, potential risks and benefits, and participants’ rights to withdraw at any time without penalty. The survey forms were filled out by the participants in approximately 15–20 min. All data has been recorded anonymously.

#### 2.3.1 Personal information form

Researchers developed a demographic information form to collect participants’ data, including age, gender, and duration of participation in recreational activities (in months).

#### 2.3.2 International physical activity questionnaire-short form

The questionnaire’s validity and reliability were assessed by Craig et al. (2003), while its Turkish adaptation was conducted by Öztürk (2005). There are a total of seven questions, each intended to assess physical activity levels over the preceding week. The form inquires about the duration individuals dedicate to vigorous and moderate physical activities, in addition to sedentary pursuits such as sitting and walking. This data is obtained by calculating metabolic equivalents (METs). The respective MET values are 1.5 for sitting, 3.3 for walking, 4 for moderate physical activity, and 8 for vigorous physical activity. Consequently, these figures facilitate the computation and appraisal of each individual’s weekly physical activity level via recurrent evaluations. Upon ascertaining the MET value, individuals’ physical activity levels can be categorized as inactive (<600 MET), minimally active (600 MET-3,000 MET), and HEPA active (>3,000 MET) (Öztürk, 2005). The Turkish version of IPAQ-SF has been shown to be both valid and reliable for assessing physical activity levels. Öztürk’s study assessed structural, concurrent, and criterion validity, along with test–retest reliability. The test–retest reliability coefficients were *r* = 0.69 for the short form and *r* = 0.64 for the long form, whereas the criterion validity coefficients were *r* = 0.30 for the short form and *r* = 0.29 for the long form. The results validate that the Turkish adaptation of IPAQ-SF yields reliable and comparable data for evaluating physical activity (Öztürk, 2005).

#### 2.3.3 Physical self-perception profile – (PSPP)

The Physical Self-Perception Profile (PSPP) developed by Fox and Corbin (1989) was used to assess individuals’ multidimensional perceptions of their physical selves. The Turkish version of the scale was adapted and validated by Aşçı et al. (1999). The PSPP consists of 30 items and includes five subdimensions, each containing six items: sport (competence), (physical) condition, body (attractiveness), strength, and physical self-worth. Sport (competence) refers to an individual’s perceived ability in sports and physical activities; (physical) condition reflects perceptions regarding endurance and fitness level; body (attractiveness) represents the individual’s perception of how attractive their body is to others; strength refers to perceived muscular strength and physical power; and physical self-worth represents the overall perception of physical adequacy related to performing movements correctly and maintaining physical endurance. Items are rated on a four-point Likert scale ranging from 1 (“most like me”) to 4 (“least like me”). Subscale scores are calculated by averaging the six items corresponding to each dimension, and the total PSPP score is obtained by averaging the five subscale scores. Higher scores indicate a higher level of perceived physical self in the relevant dimension. In the Turkish validity and reliability study conducted by Aşçı et al. (1999), Cronbach’s alpha coefficients for the subscales ranged between 0.74 and 0.88. In the present study, the Cronbach’s alpha coefficient for the total PSPP score was calculated as *α* = 0.808.

#### 2.3.4 Warwick-Edinburgh mental wellbeing Scal- short form (WEMWB-SF)

The validity and reliability tests of the scale were conducted by Stewart-Brown et al. (2009), and its adaptation into Turkish was carried out by Demirtaş and Baytemir (2019). The scale is a 5-point Likert type scale and consists of 7 items. All items in the scale consist of positive statements. The participants were asked to answer according to their recent activities in the last 2 weeks. Since the validity and reliability of the scale was carried out on Turkish university students, it is considered to be a valid and reliable measurement tool that can be used to measure the psychological well-being levels of university students. The Cronbach alpha reliability coefficient of the scale was calculated as 0.86 (Demirtaş and Baytemir, 2019).

### 2.4 Analysis of data

Data were analyzed using IBM SPSS Statistics 28.0. Prior to the main analyses, a series of preliminary analyses were conducted to examine the suitability of the data for multivariate analysis. The normality of the variables was evaluated by examining skewness and kurtosis values, following the criteria suggested by Kline (2005), where skewness values below 3 and kurtosis values below 10 indicate acceptable normality. In addition, common method bias (CMB) was assessed using Harman’s single-factor test. Furthermore, multicollinearity diagnostics were performed by examining tolerance and variance inflation factor (VIF) values. Tolerance values greater than 0.10 and VIF values below the recommended thresholds were considered indicative of the absence of multicollinearity (Hair et al., 2019).

Descriptive statistics (mean, standard deviation, minimum, maximum) have been calculated. Reliability analysis (Cronbach’s alpha) has been conducted. Pearson correlation analysis was used to examine the relationships between the variables. Group comparisons by gender were conducted using an independent samples *t*-test.

*Mediation analysis (H1 and H2):* We used PROCESS macro Model 4 (Hayes, 2013) to test how physical self-perception acts as a mediator between physical activity and psychological well-being. This model analyzed the direct impact of physical activity on psychological well-being (c′ path) and the indirect impact via physical self-perception (a × b path). We used a bootstrap method with 5,000 resamples to determine the significance of the indirect effect and calculated 95% confidence intervals (CI). Age and activity duration were included as covariates in the model to control for their potential effects.

*Analysis of moderation (H3):* Using PROCESS macro Model 7 (Hayes, 2013), we tested how gender affects the relationship between physical activity, physical self-perception, and psychological well-being. In this model, gender was identified as a moderator of the relationship between physical activity and physical self-perception.

To ensure valid multivariate analysis, this study assessed normality by examining skewness and kurtosis. Following Kline (2005), values of skewness below 3 and kurtosis below 10 are considered acceptable. The skewness values for the main variables ranged from −0.384 to 0.264, and kurtosis values ranged from −0.333 to 0.020. Therefore, all variables meet the normality criteria.

Harman’s Single Factor Test was conducted to assess common method bias (CMB) in the data. The first factor accounted for 48.121% of the variance, which is above the critical threshold of 40%–50% (Fuller et al., 2016). This suggests that there is no substantial CMB in this study.

Multicollinearity diagnostics were conducted to assess potential collinearity among the independent variables. The tolerance value was 0.864 (greater than the recommended threshold of 0.10), and the variance inflation factor (VIF) was 1.157, which is well below the conservative cutoff value of 3.3 (Hair et al., 2019). These results indicate that multicollinearity was not a concern in the present study.

## 3 Result

This section presents the results of the statistical analyzes conducted to test the research hypotheses. The analyzes include descriptive statistics, correlation analysis, group comparisons, and bootstrap-based mediation and moderation analyzes.

The average age of the participants (*N* = 468) was 20.08 (SD = 2.39), the average physical activity level was 2031.62 MET-min/week (SD = 578.29), the average psychological well-being score was 3.00 (SD = 0.76), and the average total PSPP score was 3.27 (SD = 0.68). The internal consistency reliability of the total PSPP score is *α* = 0.808, indicating a high level of reliability (Nunnally and Bernstein, 1994). According to the results of the correlation analysis, physical activity shows a positive and significant relationship with psychological well-being (*r* = 0.24, *p* < 0.001) and physical self-perception (*r* = 0.37, *p* < 0.001). The strongest relationship was observed between physical self-perception and psychological well-being (*r* = 0.61, *p* < 0.001). Among the control variables, age is positively correlated with psychological well-being (*r* = 0.17); the duration of activity shows significant positive relationships with both psychological well-being (*r* = 0.11) and physical self-perception (*r* = 0.13) (Table 1).

**Table 1:** Descriptive statistics and correlations of study variables.

Variables	M	SD	1	2	2a	2b	2c	2d	2e	3	4
1. PA	2031.6	578.29									
2. PSPP	3.27	0.68	0.37***								
2a. Sport	3.04	0.88	0.17***	0.75***							
2b. Strength	3.27	0.90	0.36***	0.74***	0.46***						
2c. Body	3.32	0.75	0.38***	0.78***	0.46***	0.46***					
2d. PSW	3.69	1.02	0.34***	0.74***	0.35***	0.41***	0.62***				
2e. Condition	3.05	0.98	0.16***	0.78***	0.58***	0.47***	0.49***	0.38***			
3. PWB	3.00	0.76	0.24***	0.61***	0.51***	0.40***	0.47***	0.38***	0.56***		
4. Age	20.08	2.39	−0.16***	−0.04	0.00	,01	−0.11*	−0.13**	0.07	0.17***	
5. AD	24.32	10.06	0.30***	0.13**	0.11*	0.11*	0.09	0.08	0.10*	0.11*	−0.03

*N* = 468. PA, physical activity, PSPP, physical self-perception profile, PSW, physical self-worth, PWB, psychological well-being; AD, activity duration. Cronbach’s alpha indicated good internal consistency (*α* = 0.808). *p < 0.05, **p < 0.01, ***p < 0.001.

The study included 274 male and 194 female participants. It was found that women (*M* = 3.42) have significantly higher physical self-perception compared to men (*M* = 3.17) (*p* < 0.001). In addition, in terms of sport, strength, and condition, women have achieved significantly higher averages compared to men. In terms of psychological well-being scores, a significant difference was also observed in favor of women (*M* = 3.09) (*p* < 0.05). In terms of physical activity levels, no significant difference was found between genders (Table 2).

**Table 2:** Comparison of variables by gender.

Variables	Male M (SD)	Female M (SD)	*t*	*p*	Cohen’s d
Physical activity	2031.39 (564.68)	2031.96 (598.45)	−0.01	0.992	0.00
PSPP	3.17 (0.71)	3.42 (0.62)	−3.93	<0.001	0.37
Sport	2.86 (0.92)	3.30 (0.76)	−5.54	<0.001	0.52
Strenght	3.18 (0.95)	3.39 (0.81)	−2.59	<0.01	0.24
Body	3.27 (0.77)	3.39 (0.70)	−1.78	0.076	0.17
PSW	3.65 (1.04)	3.75 (0.98)	−0.98	0.327	0.09
Condition	2.90 (1.01)	3.26 (0.89)	−4.00	<0.001	0.38
Psychological well-being	2.94 (0.76)	3.09 (0.74)	−2.03	0.043	0.19

PSPP, physical self-perception profile, PSW: physical self-worth, *p < 0.05, **p < 0.01, ***p < 0.001.

It has been observed that physical activity significantly predicts physical self-perception positively (a path: *β* = 0.365, *p* < 0.001). Physical self-perception has also strongly predicted psychological well-being (b path: *β* = 0.599, *p* < 0.001). While age, one of the control variables, has a significant positive effect on psychological well-being (*β* = 0.198, *p* < 0.001), the effect of activity duration is not significant. The direct effect of physical activity on psychological well-being became insignificant when the mediator variable model was added (c′ path: *β* = 0.041, *p* = 0.310). The bootstrap indirect effect was found to be significant (*β* = 0.219, 95% CI [0.151, 0.290]). These findings indicate that physical self-perception plays a full mediating role. Control variables (Age, Activity Duration) have been added as covariates to this model. That is, in the model, the effects of age and activity duration were held constant, and the main relationship was examined. Therefore, the following coefficients were reported for both dependent variables (PSPP and Psychological well-being) in the regression tables. PSPP (path a) = PA + Age + Activity Duration; Psychological well-being (paths b and c) = PA + PSPP + Age + Activity Duration (Table 3).

**Table 3:** The mediating role of Physical Self-Perception—regression coefficients including control variables.

Variables	*β*	SE	t/z	*p* / %95 CI
Dependent variable: physical self-perception (a path)				
Physical activity	0.365	0.046	7.967	<0.001
Age	0.018	0.044	0.408	0.684
Activity duration	0.020	0.045	0.445	0.657
*R*^2^ = 0.136				
Dependent variable: psychological well-being (b and c′ paths)				
Physical activity (c′ path)	0.041	0.040	1.017	0.310
Physical self-perception (b path)	0.599	0.038	15.622	<0.001
Age	0.198	0.036	5.500	<0.001
Activity duration	0.031	0.037	0.820	0.413
*R*^2^ = 0.412				
Total, direct, and indirect effects				
Total effect (c path)	0.260	0.047	5.559	<0.001
Direct effect (c′ path)	0.041	0.040	1.017	0.310
Indirect effect (a × b)	0.219	0.036	6.115	<0.001 [0.151, 0.290]

β, standardized regression coefficient; SE, standard error. For the indirect effect, 95% bootstrap confidence intervals (5,000 resamples) are presented; in this row, the *t* column represents the *z-*value, and the *p*/95% CI column indicates the confidence interval.

When examining the sub-dimension analyzes, it is observed that the strongest mediating sub-dimension is Body (*β* = 0.174). The indirect effects of all sub-dimensions were found to be statistically significant. This finding indicates that the effect of physical activity on psychological well-being operates through all dimensions of physical self-perception, albeit with varying magnitudes (see Tables 4, 5).

**Table 4:** Comparison of the mediating roles of PSPP subscales.

Mediating variables	*β*	SE	*z*	*p*	%95 CI	Significant?
Sport	0.075	0.026	2.944	0.003	[0.026, 0.129]	Yes
Strength	0.128	0.026	4.965	<0.001	[0.080, 0.181]	Yes
Body	0.174	0.03	5.777	<0.001	[0.119, 0.238]	Yes
PSW	0.118	0.022	5.299	<0.001	[0.077, 0.164]	Yes
Condition	0.081	0.028	2.929	0.003	[0.029, 0.136]	Yes

Bootstrap with 5,000 resamples. SE, bootstrap standard error.

**Table 5:** Moderating effect of gender on the mediation paths.

Interaction term	*β*	SE	*t*	*p*
Path a (PA × Gender → PSPP)	−0.122	0.086	−1.421	0.156
Path b (PSPP × Gender → PWB)	−0.004	0.077	−0.057	0.954

**Figure 2:**
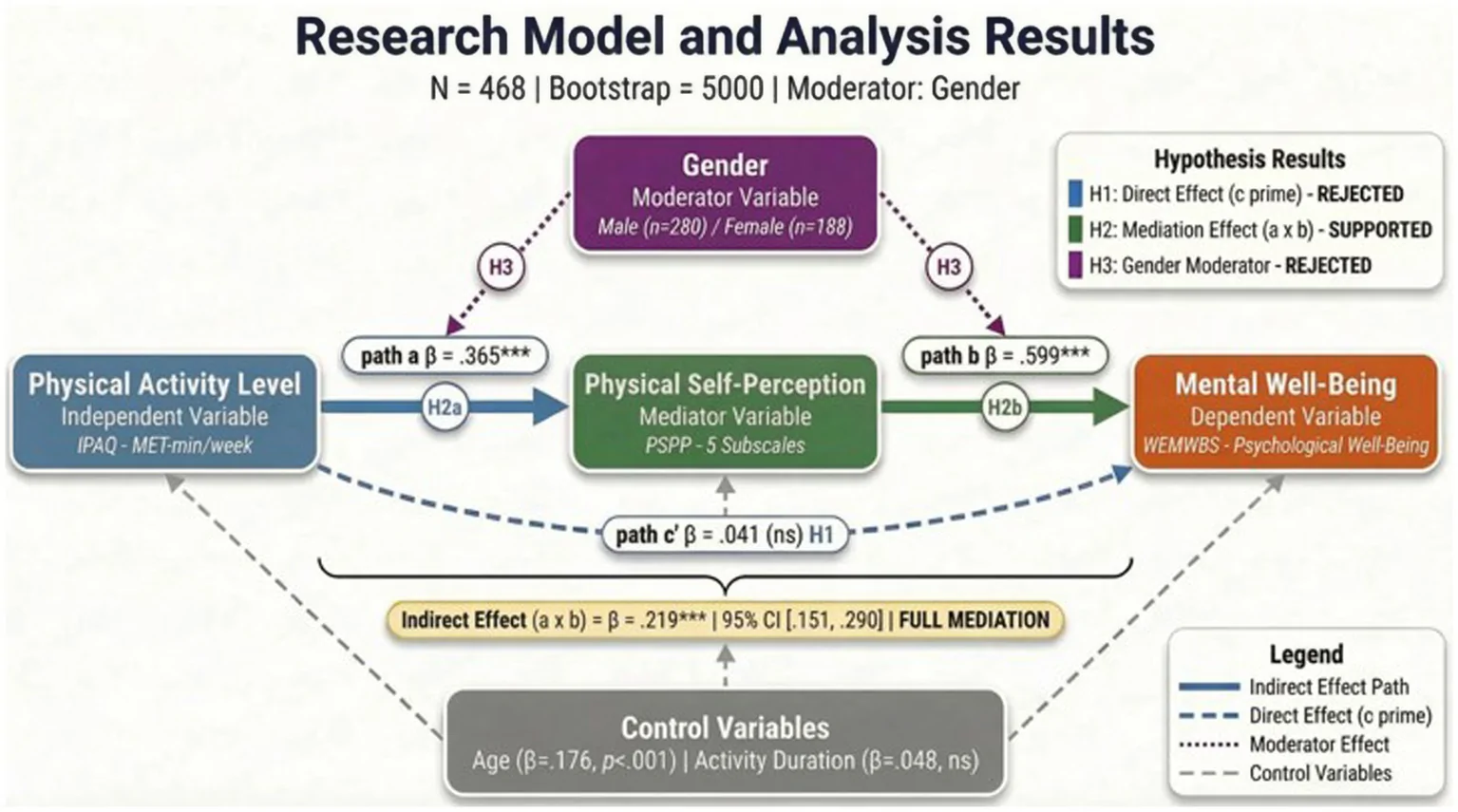
Research model and analysis results.

Moderator analysis, neither the a path (*β* = −0.122, SE = 0.086, *t* = −1.421, *p* = 0.156) nor on the b path (*β* = −0.004, SE = 0.077, *t* = −0.057, *p* = 0.954). It has shown that there is no significant moderating effect on 954. When examining the conditional indirect effects presented in Table 6, it is observed that the mediation mechanism is significant in both genders (Male: β = 0.235, 95% CI [0.152, 0.332]; Female: *β* = 0.179, 95% CI [0.083, 0.289]). However, the difference between the two groups is not statistically significant. Physical activity → physical self-perception → psychological well-being mechanism operates with similar strength in both genders (see Figure 2, Table 7).

**Table 6:** Conditional indirect effects by gender.

Gender	*β*	SE	z	*p*	%95 CI	Within-group indirect effect significant?
Male	0.235	0.045	5.169	<0.001	[0.152, 0.332]	Yes
Female	0.179	0.053	3.389	<0.001	[0.083, 0.289]	Yes

**Table 7:** Summary table of the results of the research hypotheses.

Hypothesis	Description	Key finding	Conclusion
H1	Physical activity has a direct positive effect on Psychological well-being	*β* = 0.041, *p* = 0.310	Rejected
H2	Physical self-perception mediates the relationship between physical activity and Psychological well-being.	*β* = 0.219, %95 CI [0.151, 0.290]	Supported (Full Mediation)
H3	Gender moderates the mediation model.	a yolu: *p* = 0.156; b yolu: *p* = 0.954	Rejected

The conceptual model of the study is presented in Figure 1.

*H1*: Physical activity has a direct positive effect on psychological well-being.

*H2*: Physical self-perception mediates the relationship between physical activity and psychological well-being.

*H2a*: Physical activity has a positive effect on the physical self-perception.

*H2b*: Physical self-perception has a positive effect on psychological well-being.

*H3*: Gender moderates the mediating relationship between physical activity, physical self-perception, and psychological well-being.

The research model and analysis results in Figure 2.

*H1*: Physical activity has a direct positive effect on psychological well-being.

*H2*: Physical self-perception mediates the relationship between physical activity and psychological well-being.

*H2a*: Physical activity has a positive effect on the physical self-perception.

*H2b*: Physical self-perception has a positive effect on psychological well-being.

*H3*: Gender moderates the mediating relationship between physical activity, physical self-perception, and psychological well-being.

## 4 Discussion

This study aims to investigate the mediating role of physical self-perception in the association between physical activity and psychological well-being, and the moderating role of gender in this relationship among recreational physical activity participants aged 15–24. The research findings indicate that the direct effect of physical activity on psychological well-being is not significant (H1 rejected), whereas physical self-perception plays a complete mediating role in this relationship (H2 supported). On the other hand, it has been determined that gender does not have a significant regulatory effect in this mediation model (H3 rejected). These findings are evaluated within the framework of the Conservation of Resources (COR) Theory and in light of current literature.

Below, the findings related to each hypothesis are discussed in light of the literature.

*H1*: Physical activity has a direct positive effect on psychological well-being.

The most striking finding of the research is that the impact of physical activity on psychological well-being occurs entirely through physical self-perception (full mediation). This finding is strongly consistent with the Conservation of Resources (COR) Theory. According to Hobfoll’s (1989) COR Theory, individuals strive to acquire, maintain, and enhance resources (such as emotional, cognitive, and physical) to cope with stress and preserve their psychological well-being (Hobfoll, 2011). In this context, recreational physical activity can be seen as a process in which a person proactively invests in their “resources.” Young adults aged 15 to 24 who regularly engage in less structured activities such as futsal, street basketball, or cycling, motivated by social factors, are developing positive perceptions of their bodies and constructing their physical self-concept. Wang et al. (2025) conducted a recent study demonstrating that physical activity enhances psychological resources such as self-efficacy and emotional regulation, thereby improving mental health.

The absence of a significant direct effect of physical activity on psychological well-being in this recreational sports context may be interpreted with caution from the perspective of Conservation of Resources (COR) Theory. This finding suggests that recreational physical activity can be considered a form of resource investment; however, its psychological benefits may not emerge in a direct or immediate manner. Instead, these benefits may depend, at least in part, on how individuals cognitively and emotionally appraise their physical self. From this viewpoint, the behavioral engagement in physical activity does not necessarily translate directly into improved psychological well-being outcomes. Rather, it may first contribute to the development of an important personal resource, namely a more positive physical self-perception. In this process, physical self-perception may function as a mediating resource that helps to explain how physical activity is associated with psychological well-being. This interpretation is broadly consistent with the central proposition of COR theory, which emphasizes that individuals invest resources in order to gain or protect other valued resources (Hobfoll, 2001). Within the recreational sports context, physical activity may be viewed as an initial resource investment, physical self-perception as a potential intermediate resource, and psychological well-being as a downstream outcome that may emerge through this resource-building process.

*H2*: Physical self-perception mediates the relationship between physical activity and psychological well-being.

The complete mediation finding indicates that interventions aimed at enhancing psychological well-being through physical activity should focus not only on increasing activity levels but also on fostering a positive physical self-concept. This may be achieved by providing feedback on physical progress, fostering inclusive social environments where all participants feel valued, and supporting individuals in recognizing and appreciating their physical abilities. In a comprehensive systematic review conducted by White et al. (2024), self-esteem and body image satisfaction were identified as the most significant mediating variables in the relationship between physical activity and psychological well-being. Likewise, Li and Hao (2025) conducted a study on high school students, discovering that physical activity positively influences life satisfaction and psychological well-being through the mediation of social support and self-esteem. Recreational activities, unlike structured sports, do not impose performance pressure, thereby facilitating the establishment of social connections among youth and promoting body acceptance without self-judgment. This situation is of critical importance for identity construction and body image during the transitional period from adolescence to young adulthood, specifically between the ages of 15 and 24 (Merino et al., 2024; Zhao et al., 2025; Guest et al., 2022).

The relatively stronger association observed between physical self-perception and psychological well-being compared to the association between physical activity and psychological well-being may indicate that physical self-perception plays a particularly important role in the observed relationships and highlights the need to consider alternative causal pathways. One possible explanation is that individuals who generally feel more positive about their bodies and physical abilities also tend to have better psychological well-being and engage more frequently in physical activities. From this perspective, positive physical self-perception may not necessarily emerge as a consequence of participation in recreational sports; instead, it may represent a pre-existing characteristic that simultaneously contributes to better psychological well-being and higher levels of physical activity. In other words, individuals with more positive body perceptions may form a psychologically healthier subgroup that is naturally more inclined toward physical activity, rather than physical activity directly generating improvements in physical self-perception (Ames et al., 2023; Choi et al., 2019; Wanjau et al., 2023). As noted by Sabiston et al. (2019), the predominance of cross-sectional studies examining the relationships between physical activity, sport, and body image substantially constrains our understanding of the bidirectional and reciprocal associations among these variables. This bidirectional or reverse causal pathway cannot be ruled out given the cross-sectional design of the study. Future longitudinal and experimental research is needed to establish temporal precedence and clarify whether physical activity leads to changes in physical self-perception and psychological well-being, or whether pre-existing positive physical self-perception and psychological well-being increase the likelihood of engaging in recreational sports.

Future research should also extend the present findings by conducting subgroup analyses based on different types of recreational physical activity within a Conservation of Resources (COR) Theory framework. Recreational physical activities are not homogeneous and may differ substantially in the extent to which they facilitate the acquisition, protection, or investment of personal and social resources. For example, individual activities such as walking or running may primarily contribute to intrapersonal resources, such as self-regulation and body awareness, whereas team-based recreational sports may additionally foster social resources, including belongingness and social support. Similarly, structured fitness activities may emphasize performance-related resources and competence development, while more informal recreational activities may be more strongly associated with affective restoration and stress recovery. From a COR perspective, these differences may influence the degree and pathway through which physical activity translates into physical self-perception and psychological well-being. Accordingly, examining whether the proposed mediation model varies across different activity types would provide a more nuanced understanding of resource gain processes in recreational sport settings and may help to refine the assumptions of COR Theory in applied physical activity contexts.

*H3*: Gender moderates the mediating relationship between physical activity, physical self-perception, and psychological well-being.

Some studies in the literature suggest that gender may play a moderating role in the relationships between physical activity, physical self-perception, and mental health (Luo et al., 2025; Zhao et al., 2025). However, the modest moderating effect observed in this study can be interpreted as reflecting specific characteristics of recreational sports participation. Unlike competitive or performance-oriented sports events where gender-based outcomes and social comparison processes are more pronounced, recreational physical activity is generally driven by more intrinsic and general motivations such as enjoyment, stress relief, and social stimulation. Under these conditions, the psychological meaning attributed to participation may become relatively similar for women, thus reducing potential gender differences in psychological characteristics. To support this view, Karakullukcu et al. (2025) found that among participants in amateur university sports, enjoyment of activity correlated positively with mental health in both genders, with limited evidence of differential effects. This suggests that when physical activity occurs in a non-competitive, socially inclusive environment, psychological relationships may emerge more homogeneously across gender groups. Consequently, the age range of this sample (15–24 years) and its recreational nature may have contributed to the formation of a context in which gender-specific socialization patterns and expectations regarding sports were present. This may help explain why gender is not a significant moderating factor in the proposed model.

## 5 Conclusion

The results of this study indicate that physical self-perception is a significant factor influencing the psychological well-being of adolescents in recreational sports settings. Consequently, it is imperative for coaches and recreational sport programs to deliver constructive feedback regarding athletes’ physical development, foster inclusive and supportive sports environments, and emphasize individual development. Psychological well-being professionals should help people develop a positive and realistic sense of self by focusing on their physical abilities through physical activity-based interventions. Public health policies should not only encourage participation in physical activity but also support inclusive and accessible sport programs that enhance individuals’ physical self-perception. Recreational sports programs should be designed to provide equal participation opportunities for both women and men, and they should support fair and inclusive access to sports environments for all individuals.

Based on the findings and limitations, the following recommendations can be made for future research: Subsequent research may employ longitudinal designs or randomized controlled trials (RCTs) to examine the effects of recreational physical activity interventions on physical self-perception and psychological well-being over time. To better understand the relationship between physical activity and psychological well-being, it would be beneficial to test other psychological resources such as social support, self-efficacy, psychological resilience, and emotion regulation in chain mediation models (Wang et al., 2025; Tang et al., 2025). Utilizing objective measurement tools to ascertain levels of physical activity will enhance the reliability of the data.

### 5.1 Limitations of the study

When interpreting the findings of this study, several limitations should be acknowledged. First, the absence of a sedentary control group limits the ability to determine whether the observed associations are specific to recreational physical activity or reflect pre-existing characteristics of already active individuals. Consequently, the findings cannot be generalized to inactive populations, and future studies should include sedentary comparison groups to strengthen causal inference regarding the effects of physical activity. Second, the cross-sectional design precludes causal interpretation. Although the mediation model suggests that physical activity is linked to higher physical self-perception, which in turn relates to better psychological well-being, temporal ordering cannot be established. Reverse and bidirectional relationships are also plausible; for example, individuals with more positive physical self-perceptions may be more likely to engage in physical activity. Longitudinal and experimental designs are therefore required to clarify directionality. Third, convenience sampling was used, with participants recruited at selected recreational sports venues during specific times. This approach may have introduced selection bias and limits representativeness with respect to socioeconomic status, geographic diversity, and activity preferences. In addition, self-selection into research participation may further reduce external validity, limiting generalization beyond urban recreational sport participants. Fourth, all key variables, including physical activity (IPAQ) and psychological well-being outcomes, were assessed via self-report, which may be affected by recall bias and social desirability effects. Finally, the sample was restricted to individuals aged 15–24 years who regularly participate in recreational physical activity, which limits generalizability to other age groups and populations with different activity profiles.

## Data Availability

The raw data supporting the conclusions of this article will be made available by the authors, without undue reservation.
